# Long-term dynamics of placozoan culture: emerging models for population and space biology

**DOI:** 10.3389/fcell.2024.1514553

**Published:** 2025-01-08

**Authors:** Daria Y. Romanova, Alexander A. Povernov, Mikhail A. Nikitin, Simkha I. Borman, Yana A. Frank, Leonid L. Moroz

**Affiliations:** ^1^ Lab of Cellular Neurobiology of Learning, Institute of Higher Nervous Activity and Neurophysiology of RAS, Moscow, Russia; ^2^ Koltzov Institute of Developmental Biology of Russian Academy of Sciences, Moscow, Russia; ^3^ Departments of Neuroscience and McKnight Brain Institute, University of Florida, Gainesville, FL, United States; ^4^ Whitney Laboratory for Marine Bioscience, University of Florida, Gainesville, FL, United States

**Keywords:** Placozoa, *Trichoplax*, long-term culturing, aging of culture, behavior, space biology

## Abstract

As the simplest free-living animal, *Trichoplax adhaerens* (Placozoa) is emerging as a powerful paradigm to decipher molecular and cellular bases of behavior, enabling integrative studies at all levels of biological organization in the context of metazoan evolution and parallel origins of neural organization. However, the progress in this direction also depends on the ability to maintain a long-term culture of placozoans. Here, we report the dynamic of *Trichoplax* cultures over 11 years of observations from a starting clonal line, including 7 years of culturing under antibiotic (ampicillin) treatment. This study revealed very complex population dynamics, with seasonal oscillation and at least partial correlations with the solar radio emission flux and the magnetic field disturbance parameters. Notable, the analysis of the distribution of Fe^2+^ in living animals revealed not only its high abundance across most cells but also asymmetric localizations of Fe^2+^ in unidentified cells, suggesting that these Fe^2+^ intracellular patterns might be coupled with the animal’s bioenergetics. We hypothesize that placozoans might have magnetoreception, which can be experimentally tested in future studies. In sum, *Trichoplax*, in particular, and Placozoa, in general, can be viewed as prospective reference species in traditional evolutionary and system biology but have the yet unexplored potential for planetary ecology and space biomedicine.

## 1 Introduction

Placozoans are one of the most ancient branches of metazoans. These animals have a simple bodyplan with a dozen of cell types (Smith et al., 2014). The phenotypic similarity of placozoan haplotypes and species provides the dissonance between nearly identical morphological architectures and substantial genetic distances when comparing their genomes (Srivastava et al., 2008) and transcriptomes. Mechanisms of complex behavior repertoirs with elements of social interactions between individuals (Kuhl and Kuhl, 1963; Kuhl and Kuhl, 1966; Seravin and Karpenko, 1987; Seravin and Gudkov, 2005a; Seravin and Gudkov, 2005b; Eitel and Schierwater, 2010; Eitel et al., 2013; Smith et al., 2015; Senatore et al., 2017; Armon et al., 2018; Zuccolotto-Arellano and Cuervo-González, 2020; Zhong et al., 2023) requires a detailed comparative analysis of cellular and system features, ideally for all proposed placozoan species/haplotypes (Miyazawa et al., 2012; Eitel, 2010; Eitel et al., 2013; Aleshin et al., 2004; Miyazawa et al., 2021; Schierwater and DeSalle, 2018; Tessler et al., 2022). However, whether there are species-specific growth dynamics is still an open question; and this information can be essential for taxonomy and speciation.

Placozoa inhabit temperate to tropical latitudes (Ueda et al., 1999; Signorovitch et al., 2006; Pearse and Voigt, 2007; Eitel and Schierwater, 2010; Nakano, 2014), preferring calm coastal waters over a wide range of salinities (Pearse, 1989), depths (Eitel et al., 2013), pH (Schleicherová et al., 2017; Schierwater, 2005; Schierwater et al., 2010), and temperatures (Pearse, 1989). The animals are bottom dwellers on substrates of a mixture of bacteria and algae (Grell, 1972; Ruthmann, 1977; Signorovitch et al., 2006; Seravin and Gerasimova, 1998; Jackson and Buss, 2009; Smith et al., 2014). At present, the ecological niches and exact food sources for Placozoa are only loosely defined. Pearse and Voigt (2007) suggested that Placozoa in tropical and subtropical areas feed by consuming organic detritus, as well as algae and bacteria that form biofilms on corals and rocky bottoms.

Today, most experimental work on Placozoa is carried out under conditions of culturing, comparable to their natural microenvironments in biotopes: photoperiod or climate control at 6:18 h, temperature of 22–24° Celsius in Petri dishes (Heyland et al., 2014; Romanova et al., 2022; Romanova et al., 2024). Since *in situ* observations on placozoans are difficult to perform, there are small numbers of identified environmental parameters in their natural habitats.

It has been shown that, depending on the quality and quantity of the substrate, animals can exhibit three patterns of population dynamics under cultured conditions (Romanova et al., 2022): 1) with a sufficient density of microalgae, animals are capable of dividing once a day or two, 2) with a lack of food substrate, a decrease in the animal biomass and body sizes had been observed, and asexual reproduction might be suppressed, 3) a significant increase in the biomass of animals and the appearance of aggregations of animals in Petri dishes occurred on a thick substrate of green algae *T. marina*, as the primary food source.

Assessing the population size is straightforward since animals reproduce asexualy, gaining biomass in cell culture (Okshtein, 1987). Sexual reproduction in laboratory conditions has been rarely observed, and only early stages of development (up to 128 cells) have been reported (Eitel et al., 2011). Given a high animal density and food scarcity as possibe indictor of embryo formation (Eitel et al., 2011), sexual reproduction may not contribute to population growth in laboratory conditions.

Long-term culturing in laboratory conditions, as well as field observations, have shown that there is a seasonal dependence on the number of individuals in populations over the years (Ueda et al., 1999; Maruyama, 2004; Pearse and Voigt, 2007), related to changes in illumination, temperature, and chemical parameters of the environment such as acidity (Schleicherová et al., 2017). And in natural conditions population dynamics might be associated with complex biofilm dynamics, light and temperature gradients, as well as tides and hydodynamics (Pearse and Voigt, 2007).

Here, we report the seasonal changes in population growth rates of *Trichoplax adhaerens* (Placozoa) under constant temperature conditions over an 11-year observation period from January 2014 to December 2024.

## 2 Material and methods

### 2.1 Culturing of placozoans

We used axenic clonal cultures of the major placozoan reference species: *Trichoplax adhaerens* (Grell’s strain Н1, from the Red Sea). We maintained all populations in culture for 11 years (2014–2024), allowing long-term observations and adjustments of culture conditions (Romanova et al., 2022; Romanova et al., 2024).

A suspension of the green alga *Tetraselmis marina* (WoRMS Aphia, ID 376158) was added to the culture dishes. When the biofilm of microalgae became thinner or depleted, freshly prepared 1–2 mL suspension of *T. marina* could be added to the culture dishes weekly (∼6.5 × 10^6^ cells of *T. marina* per 1 μL). H1 haplotype was maintained at the two environmental chambers. The first option is a room with controlled ventilation, constant temperature (24°), and humidity (∼70%), natural light (comes from windows). The second option is a climate chamber (#B3, Europolitest), with a photoperiod of 6:18, lighting of 400 lux, and temperature of 24°C.

Under long-term culturing, animals usually divided every 1–2 days without signs of sexual reproduction (Malakhov, 1990; Zuccolotto-Arellano and Cuervo-González, 2020).

### 2.2 Individual treatment with antibiotics

*Trichoplax* might contain potentially symbiotic bacteria in fiber cells (Driscoll et al., 2013; Kamm et al., 2019). To control levels of potential bacterial endosymbionts and their effect on culturing dynamics, we used treatment with different antibiotics [ampicillin (5 μg/mL), doxycycline (1.25 μg/mL)]. Three groups are: control clonal line (CCL), ampicillin clonal line (ACL), and doxycycline clonal line (DCL).

To control the efficiency of antibiotic treatments, total DNA from individual animals was extracted using a silica-based DiaTom DNAprep 100 kit (Isogene, Moscow, Russia) according to the manufacturer’s protocol. Amplification was performed using EncycloPlus PCR kit (Evrogen, Moscow, Russia) with the following program: 95°C – 3 min, 35 cycles of PCR (95°C – 20 s, 50°C – 20s, 72°C – 1 min), and 72°C – 5 min. We have used universal forward primer 27F (AGA GTT TGA TCM TGG CTC AG) and specific reverse primer 449R (ACC GTC ATT ATC TTC YCC AC). The reverse primer was designed against 16S RNA of *Rickettsia belli* (NR_074484.2) and sequences from *Trichoplax* DNA were obtained through NCBI Trace Archive Blast using NR_074484.2 as the query. After 6 months of ampicillin treatment, the markers of *Rickettsia* were not detected.

### 2.3 F10.7 and ap indices

The solar activity index is the radio emission flux F10.7 with a wavelength of 10.7 cm (2,800 MHz). It is measured in solar flux units: 1 s.f.u. = 10^−22^ W/(m^2^*Hz). The data are presented as observed values measured by a solar radio telescope (https://www.ngdc.noaa.gov/stp/solar/solar-indices.html).

The Kp index characterizes the global disturbance of the Earth’s magnetic field in a three-hour time interval. The Kp index is defined as the average value of the disturbance levels of two horizontal components of the geomagnetic field observed at 13 selected magnetic observatories located in the subauroral zone between 48 and 63° north and south geomagnetic latitudes. The ap index is calculated from the Kp index values and represents the change in the most disturbed element of the magnetic field D or H in a three-hour time interval at mid-latitude stations. The ap index is called the planetary amplitude in a three-hour interval. The ap index varies in the range from 0 to 400 and represents the Kp values converted to a linear scale in nanoteslas (nT), which shows the equivalent amplitude disturbance at the station.

The correlation results presented in this paper rely on geomagnetic indices made available by ISGI Collaborating Institutes from data collected at magnetic observatories. We thank the involved national institutes, the INTERMAGNET network and ISGI (isgi.unistra.fr).

### 2.4 Statistical analysis

For population growth rate (PGR) experiments, we cultured axenic lines of H1 haplotype at constant temperature (24°С) and natural light in environmental chambers for 11 years. PGR, locomotion, number of individuals, and occurrences of aggregates were monitored every third day at the same time as counts of animals.

We used the Statistica software 7.0 (Afifi et al., 2003). The Kolmogorov-Smirnov criterion was used to test a hypotheses about cut belongings of parameter samples. The Spearman rank correlation test and the Pearson goodness-of-fit test were used to study the correlation between samples. These methods were applied both to cut samples as a whole and their grouping for animals of control, ampicillin, and doxycycline clonal lines. To detect differences between groups, Kruskall-Wallis ANOVA was used to compare all three samples simultaneously, and Mann-Whitney U-test was used to compare pairwise samples.

To make cut lines on the graph smoother and easier to perceive, as well as to show longer trends in cut changes in number of animals, ap and F10.7 indices, a moving average over a fixed number of observations of size 5 for each cup was shown. This number was chosen for the reason that a smaller window size did not significantly change the resulting image, and larger sizes had a noticeable effect on the amount of total displayed data. To find confidence intervals, the variance and standard deviation in numbers of animals, ap and F10.7 indices over a 5-day interval for each cup were calculated, and median values were found. The boundaries of the confidence interval were calculated as the sum and difference of the median and the standard deviations.

For visualizing and plotting, we used Pandas, Matplotlib, and Seaborn in a Python environment by Anaconda-Navigator (JupiterLab).

### 2.5 Staining

Before fixation, a dozen of individuals were allowed to settle on the bottom for one hour in 35 mm Petri dish. Then we add 0.5–1 µL of 1 mM HMRhoNox-M dye (#3317-50 μg, LumiProbe, Russia) in one hour. Fixation was achieved by gently adding 30 µL of 0.25% glutaraldehyde in artificial seawater (ASW) at room temperature for 10 min. Next, preparations were washed in phosphate buffer solution (0.1 M, pH = 7.4) three times (20 min) and mounted on a slide using Prolong gold antifade reagent with DAPI, and stored in the dark at 4°C. The samples were examined using a laser confocal Cerna-based microscope (Thorlabs, United States). Image processing was carried out using ImageJ software.

## 3 Results

### 3.1 Long-term culturing for eleven years

**FIGURE 1:**
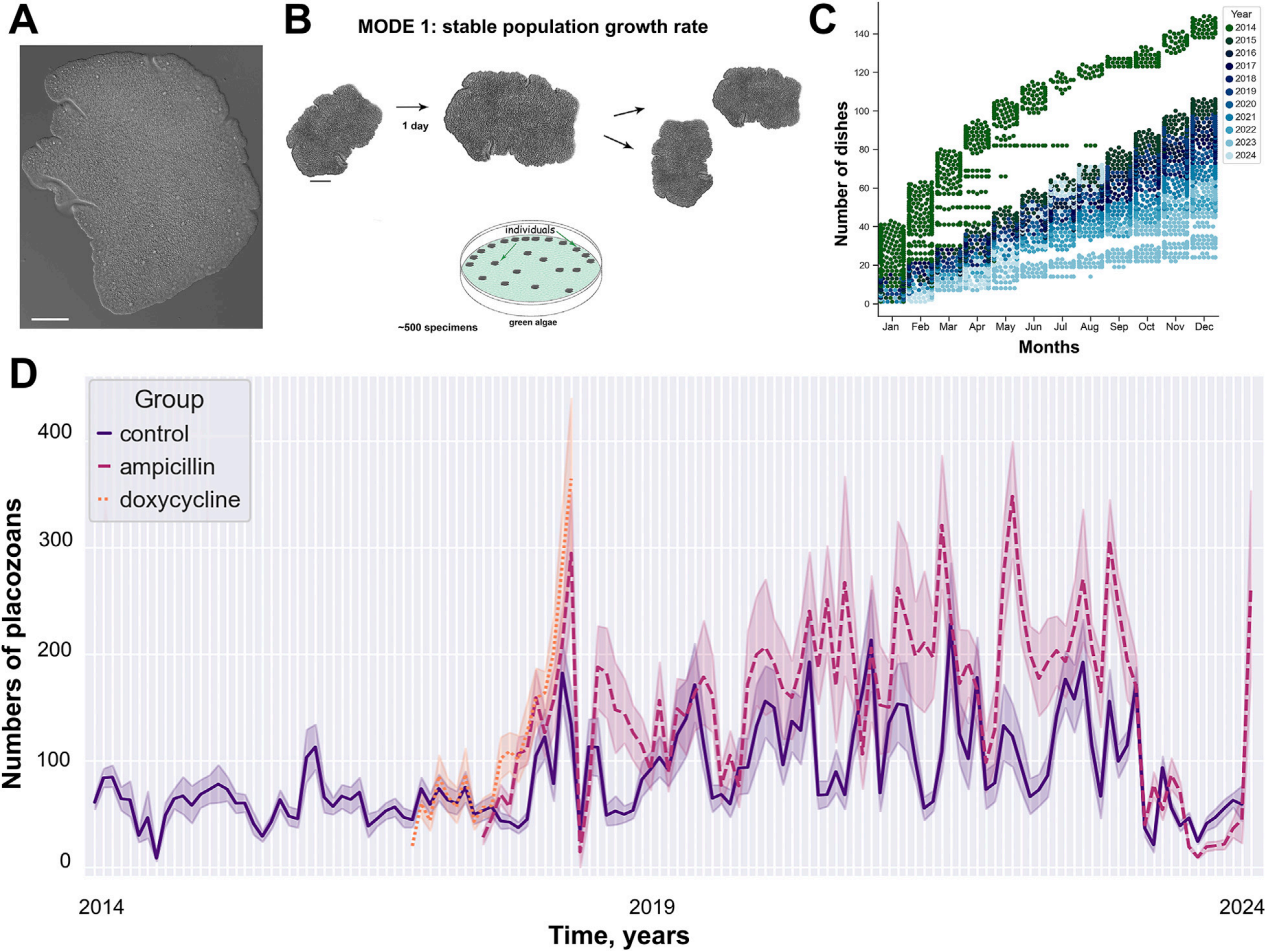
11 years of long-term culturing of placozoans. **(A)** Photo of Trichoplax adhaerens, H1 haplotype **(B)** Mode 1: stable population growth rate under culture condition. **(C)** The number of Petri dishes (earch dot represents one dish, colors mark different years) as used in the study. **(D)** Oscillations of the population growth (meant +/− standard errors) under three exparimental conditions (control/CCL, ampicillin/ACL, and doxycycline/DCL). See details in Supplementary Table S1. **(B)** modified from Romanova et al. (2022).

**FIGURE 2:**
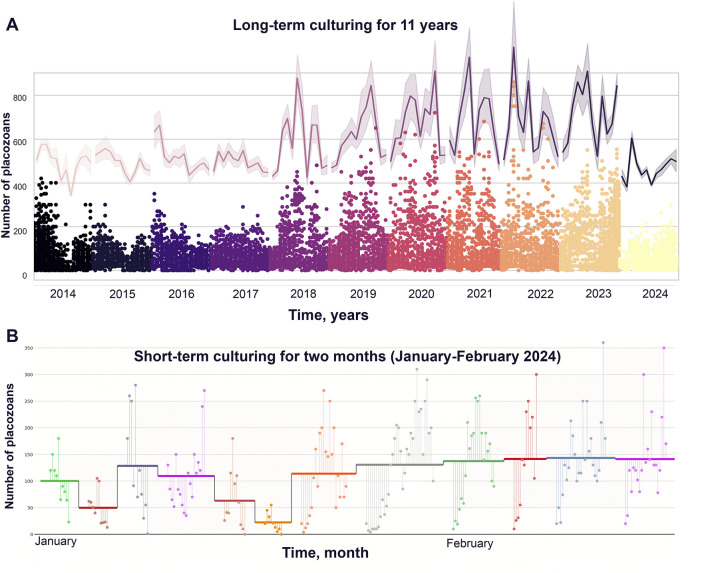
Annual and monthly population dynamics during long-term cultivation of *Trichoplax adhaerens*. **(A)** Long-term culturing from 2014 to 2024 (up to December). Oscillations of the population growth (meant +/− standard errors); continuous error bands are a graphical representation of error or uncertainty shown as a shaded region around a main trace (upper purple line in the gradient graph (the same in in Figure 1D) in gradient graph). **(B)** Dynamic of culturing during January-February 2024. On the graph: all colors–sequential Petri dishes, horizontal line for every dish is mean values of animals’ count (mean +/− standard error).

The analysis of animal population growth rates (PGR) from 2014 to 2024 showed unspecified oscillation in the dynamics of *Trichoplax* populations for the optimized culture protocol (Figure 1A; Supplementary Table S1). Weekly, freshly prepared 1–2 mL suspension of *T. marina* was be added to the culture dishes. Throughout the observation period, animals were kept under uniform conditions: constant temperature of 24°C, natural illumination, shaded from direct sunlight, regular change of artificial seawater (twice a week). Population dynamics showed repetitive changes for annual (Figure 2A) and monthly (Figure 2B) intervals.

### 3.2 Antibiotic clonal lines

Antibiotic tests were chosen as additional parameters to detect long-term changes in the PGR dynamics of *Trichoplax* relative to the external and internal microbiomes, and theirs potential impacts on structures of placozoan’s populations. Long-term observation data for the test groups with ampicillin and doxycycline showed an increase in the growth of the number of animals relative to the control (Figure 2).

There is a statistically significant difference both for the sampling over all years (p < 0.001 in K-W ANOVA, p < 0.001 in M-W U-test between all groups) and for each year separately: from 2018 to 2023 between the control and ampicillin groups (p < 0.001 in M-W U-test), between the control and doxycycline groups in 2017 (p = 0.011) and 2018 (p < 0.001), as well as between the ampicillin and doxycycline groups in 2018 (p = 0.017). In 2017 and 2024, no statistically significant differences were found between the number of animals in the control and ampicillin groups (p = 0.665, p = 0.767, respectively). Statistically significant mounthly differences between the control and ampicillin were found in 53 months out of 85 (Supplementary Table S2): 3 out of 4 months in 2017, 9 out of 12 months in 2018, 5 out of 12 months in 2019 and 2020, 8 out of 12 months in 2021, 7 out of 12 in 2022, 9 out of 12 in 2023, 7 out of 9 in 2024 (p < 0.05, M-W U-test). The descriptive statistics parameters (Median, [LQ, UQ]) for animals in the control group are 52, [23; 112], while for animals of the ampicillin group – 95, [30; 220] with the number of observations being 9203 and 4419, respectively.

Based on the fact that the groups with ampicillin and doxycycline have an increase in the growth compare to the control group, combined with the presence of statistically significant differences between the groups, we can assume that populations treated with ampicillin and doxycycline are more stable, due to the suppression of both external microbiomes and symbionts.

### 3.3 Potential planetary-scale factors and population dynamics

**FIGURE 3:**
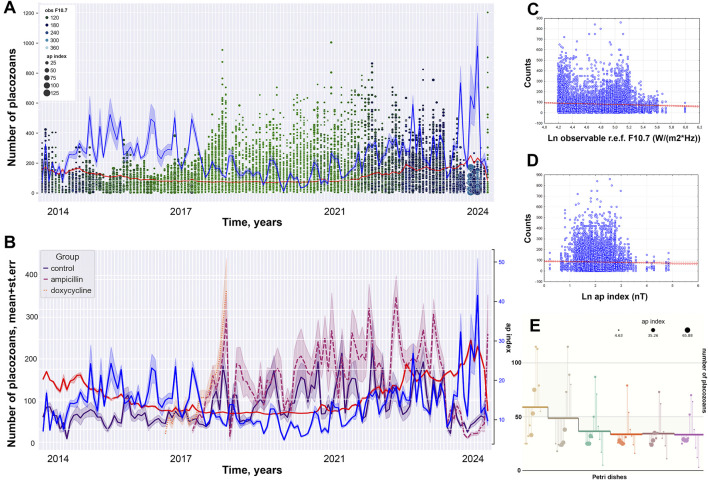
Decade of oscillation in population growth rate (PGR) (control group). **(A)** Long-term culturing of control clonal individuals for 11 years (dot represent numbers of animal per a dish). **(B)** Dynamic of PGR for normal clonal live and antibiotic treatment groups with ap and F10.7 indices (blue and red lines respectively). **(C)** Pearson scatterplot for the observable solar F10.7 index versus numbers of placozoans. **(D)** Pearson scatterplot for the ap index versus numbers of placozoans. **(E)** Numbers of animals in the individual Petri dishes during 2024 overlaped with data of ap index for the current period.

Here, we noted potential correlations of the observed long-term culture dynamics with the intensity of solar activity factors and disturbance of the magnetic field of Earth (i.e., the ap and F10.7 indices) similar to reports on other organisms (Häder, 2000; Deng et al., 2018; Neale and Thomas, 2016). According to the Kolmogorov-Smirnov criterion, the values of PGR data and the ap and F10.7 indices are not normally distributed at a significance level of <0.01. The Pearson correlation coefficient between the ap and F10.7 indices was equal to 0.27 at a significance level of p < 0.001, which hints a potential tendency for a joint increase or decrease in the values of these parameters (Figure 3).

Pearson correlation coefficients between the number of animals and the ap and F10.7 indices were equal to −0.05 (p< 0.001) and −0.03 (p = 0.004), which indicates a statistically significant weak negative effect of high values of these parameters on the number of animals. Spearman correlation coefficient between the total number for 11 years and the F10.7 index was equal to −0.06 (p < 0.001), which also indicates a statistically significant weak negative effect of high values of this parameter on the growth of the number of animals, while the correlation coefficient of the ap index is equal to 0.014 (p = 0.193). The values of the correlation coefficients between the number of animals and the ap index in the annual analysis have low levels of reliability. However, when comparing data by months and weeks of observation between groups, statistical reliability has a support at the corridor of values of p < 0.05–0.001. The low levels of reliability of the correlation coefficients between the number of animals and the ap index in the annual analysis and the levels in the study by month and weeks might be observed due to the fact that the values of the ap index differ greatly between years of observation, and slightly differ between weeks and months.

When we grouped the datasets by the ap index, the influence of the F10.7 index on the number of animals changes from negative to positive with an increase in the ap index values: the Pearson correlation coefficient is −0.112 (p = 0.116) for the range of 0–2 nT; −0.12 (p < 0.001) for the range of 2–4 nT; −0.11 (p < 0.001) for the range of 4–6 nT; −0.115 (p < 0.001) for the range of 6–8 nT; −0.043 (p = 0.113) for the range of 8–10 nT; 0.088 (p = 0.024) for the range of 10–12 nT; 0.129 (p = 0.007) for the range 12–14 nT and 0.049 (p = 0.112) for the range >14 nT. This implies a statistically significant negative correlation of solar activity at low values of the ap index, and a possible positive impact at average values.

The effect of the ap index on animal abundance also varies, showing a negative impact at low and high values of the radiation flux and a positive one at medium values: at solar activity values equal to 60–80 s.f.u. (−0.061 at p < 0.001), 80–100 s.f.u. (−0.116 at p < 0.001), 100–120 s.f.u. (−0.075 at p = 0.026) and 200–220 s.f.u. (−0.075 at p = 0.797). For the range of radiation flux values of 120–140 s.f.u. (Pc = 0.002 at *p* = 0.955), 140–160 s.f.u. (Pc = 0.086 at p = 0.003), 160–180 s.f.u. (Pc = 0.336 at p< 0.001) and 180–200 s.f.u. (Pc = 0.042; p < 0.001), the Pearson correlation coefficients were calculated. This can be infered as statistically significant negative correlation of magnetic field disturbances at low and high values of the solar radio flux, as well as a positive effect at average values.

The Spearman correlation coefficient for groupings between the number of animals and solar activity for grouping geomagnetic activity was −0.048 (p = 0.502) for the 0–2 nT group, −0.137 (p < 0.001) for the 2–4 nT group, −0.141 (p < 0.001) for the 4–6 nT group, −0.079 (p = 0.001) for the 6–8 nT group, −0.031 (p = 0.254) for the 8–10 nT group, 0.086 (p = 0.028) for the 10–12 nT group, 0.193 (p < 0.001) for the 12–14 nT group, and 0.039 (*p* = 0.209) for the >14 nT group. This implies a statistically significant negative correlation of solar activity at low values of the ap index, and a positive correlation at average values.

The Spearman correlation coefficient for groupings between the number of animals and the ap index with the grouping index F10.7 was −0.033 (p = 0.055) for the 60–80 s.f.u. group, −0.118 (p < 0.001) for the 80–100 s.f.u. group, 0.023 (p = 0.499) for the 100–120 s.f.u. group, 0.042 (p = 0.192) for the 120–140 s.f.u. group, 0.09 (p = 0.002) for the 140–160 s.f.u. group, 0.393 (p < 0.001) for the 160–180 s.f.u. group, 0.136 (p = 0.007) for the 180–200 s.f.u., 0.35 (p < 0.001) for the 200–220 s.f.u. group and 0.055 (p = 0.55) for the >220 s.f.u. group. This suggests negative correlation of magnetic field disturbances at low values of the solar radio flux, as well as a positive correlation at average values. Thus, we can hypothesize that there is a negative correlation of low values of the F10.7 index on population growth in *Trichoplax*, and a positive correlation at average values, and the correlation of the ap index is positive at average values, and negative at low and high values.

When studying the scatter diagrams, two peaks in the number of animals are observed—at low and medium-high values of the F10.7 index: 60–80 s.f.u. (Sp. R = −0.044 (p = 0.012)) and 140–160 s.f.u. (Sp. R = −0.067 (p = 0.021)); and a sharp decline at very high values of the F10.7 index: 200–220 s.f.u. (Sp. R = −0.108 (p = 0.096)) and >220 s.f.u. (Sp. R = −0.06 (p = 0.479)). For the ap index, the peak in abundance is observed at its average values: 8–10 nT (Sp. R = 0.026 (p = 0.304)), 10–12 nT (Sp. R = -0.043 (p = 0.22)), 12–14 nT (Sp. R = 0.126 (p = 0.001)) and 12–14 nT (Sp. R = 0.215 (p < 0.001); declines in abundance occur at very low and very high values: <2 nT (Sp. R = −0.014 (p = 0.939)), 2–4 nT (Sp. R = −0.002 (p = 0.937)), >20 nT (Sp. R = −0.1 (p = 0.059)).

Based on the results obtained, we can suggest that the influence of the F10.7 index on population growth in *Trichoplax* would be negative at low values and positive at average values, and the influence of the ap index is negative at low and high values and positive at average values. Therefore, we can hypothesize that an optimal range of values of the F10.7 and ap indices potentially contributing to population growth in *Trichoplax*, and the values of the indices are approximately as follows: F10.7–140–200 s.f.u., ap – 10–16 nT.

### 3.4 Fe^2+^-selective staining of individuals

**FIGURE 4:**
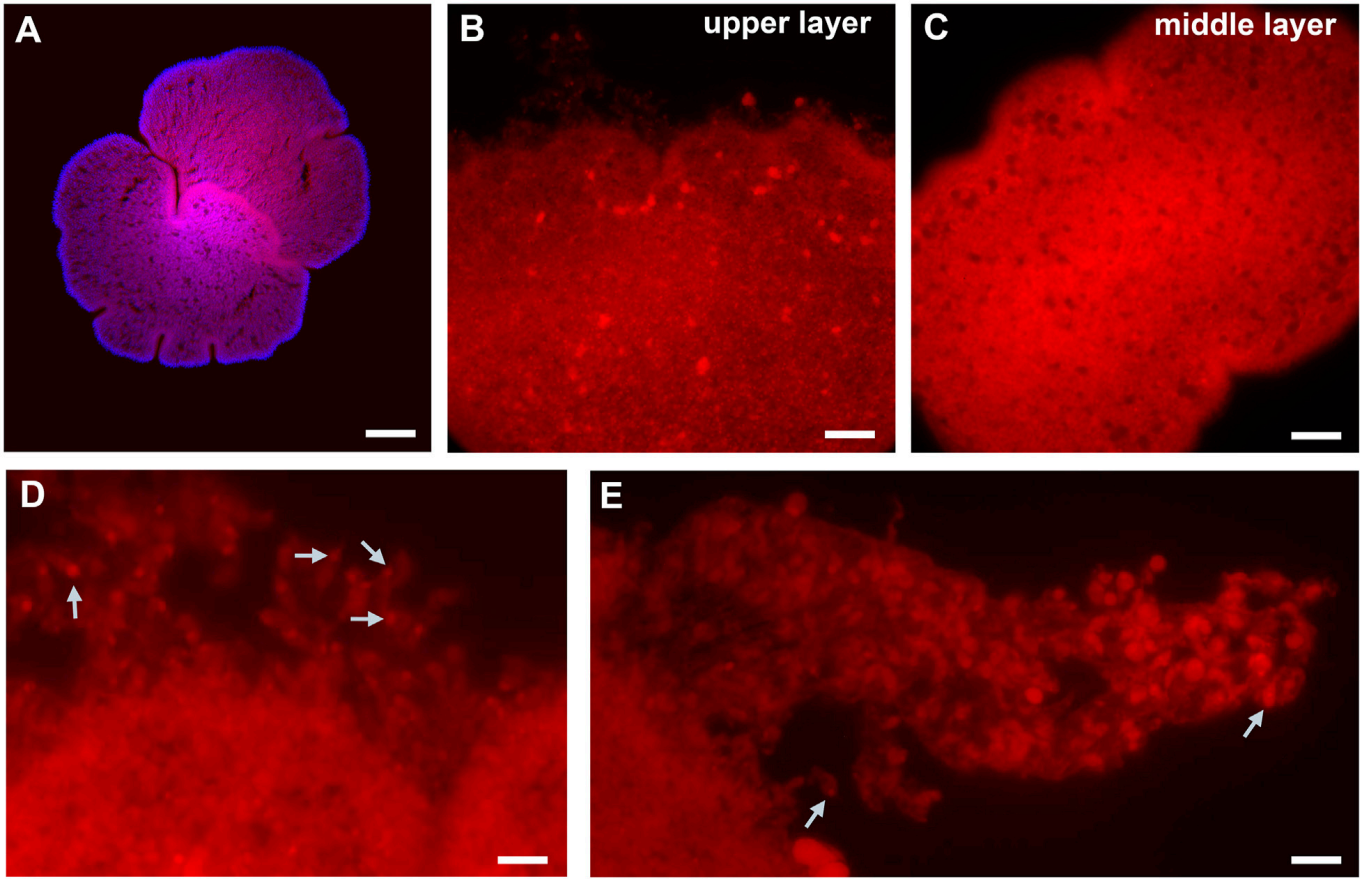
Fe^2+^-selective staining in Trichoplax. **(A)** Top view Red: HMRhoNox-M, blue–DAPI. **(B)** Focus on upper epithelia with large inclusions, and damaged rim [high magnification, see **(D)**]. **(C)** Middle layer focus with microcavities. **(D)** Rim area: epithelial cells with fluorescent signal inside. **(E)** The part of damaged rim epithelium: some cells show assymetric distribution of fluorescent signal location. White arrows point to areas of inclusions presumably enriched in Fe^2+^. Scale bar: **(A)**-100 µm, **(B)**-20 µm, **(C)**-50 µm, **(D, E)** – 10 µm.

To initially assess the cellular bases of possible geomagnetic effects, we used a Fe^2+^-selective fluorescent indicator to visualize the distribution of iron ions in lysosomes (Lv and Shang, 2018; Hirayama et al., 2019). Figure 4 shows widespread labeling with the dye, suggesting the abundant and broad distribution of Fe^2+^ across most cell types. The most intense labeling was observed in the “digestive” zone, where lipophilic, secretory, and glandular cell types, involved in the nutrition process, are located. The edge of the epithelium zone was stained significantly less than the “digestive” zone. Finally, for large, unidentified cell types, we detected asymmetrical localization of Fe^2+^ (Figure 4B); labeling occurred outside nuclei as an organelle-like localization, similar to the morphology of crystal cells (although these were not crystal cells).

## 4 Discussion

Since placozoans are an integral part of marine ecosystems (Schierwater et al., 2010; Schierwater et al., 2021; Nakano, 2014) and involved in benthic food chains, it is important to assess the parameters associated with the growth of animal populations. Any population has internal and external factors that impact its structure and survival, such as seasonal temperature fluctuations, oceanic currents, dynamics of biogenic micro- and macroelements, and even solar activity (Chizhevsky, 1976; Ranta et al., 1997; Kravchenko et al., 2006). It was also reported that temperature and pH could significantly affect the physiology of placozoans (Schleicherová et al., 2017; Schierwater, 2005; Schierwater et al., 2010), and algal substrates can modulate the behavior of both individual animals and the population as a whole (Romanova et al., 2022).

Under favorable conditions, animals divide every 1–2 days (Heyland et al., 2014; Romanova et al., 2024; Okshtein, 1987). Such a steady increase in cell numbers (due to mitosis) and size of *Trichoplax* following subsequent asexual reproduction by division is an excellent model for assessing the growth of an animal population under long-term influences influence of external factors. Here, the use of standardized, well-controlled parameters allowed us to reveal complex oscillations in population growth, with potentially dynamic regulation of the number of individuals during monthly, seasonal, and annual observation periods over eleven years (2014–2024, Supplementary Figures S1, S2, S4). Notable, the evident correlation between the population growth in placozoans and solar activity can be depicted from long-term observations independently performed by Maruyama (Maruyama, 2004); see Supplementary Figure S3 for details.

Wave-like dynamics of the number of *Trichoplax* under constant cultivation conditions allowed us to consider additional external factors potentially influencing the animal population growth. We hypothesize that two external factors have effects on the PGR - the solar radio emission flux with a wavelength of 10.7 cm/2,800 MHz (by index F10.7) and the magnetic field disturbance parameter in a three-hour time interval (by ap index) with a degree of reliability when analyzing the studied groups within each year of observation. This should be a subject of careful future investigations. Ideal controls, in the future, can be performed in magnetic chambers or as another long-term project at different geographic locations, ideally with international collaboration.

In the quest for the potential presence and mechanisms of magnetoreception in placozoans, we found that the highly selective indicator for Fe^2+^ revealed the abundance of iron in nearly every placozoan cell, with the predominant localization in the digestive zone. Some links to the physiology of Placozoa might be associated with endosymbiotic bacteria (Kamm et al., 2019), again to be investigated in the future.

It is known that bacterial cells can have magnetosomes, which are organelles made of magnetite (Fe_3_O_4_) or greigite (Fe_3_S_4_) with a phospholipid bilayer and associated proteins MamK/MamY, Ccfm (Pfeiffer et al., 2020; Liu et al., 2024). According to this hypothesis about the nature of magnetosomes, they might be associated with vesicles as a storage system for ferrous iron ions (Lv and Shang, 2018) and serve as a potential exaptation to sense magnetic fields. Whether such a system exists in endosymbiotic bacteria or specialized cells of placozoans needs to be determined in future studies.

## 5 Conclusions and future directions

We report the complex oscillations within *Trichoplax* populations during over 11 years of culturing. This study also implies a very complex population dynamics and likely partial correlations with the solar radio emission flux and the magnetic field disturbance parameters.

Notable, the analysis of the distribution of Fe^2+^ in living placozoans illuminated not only its high abundance across most cells but also asymmetric localizations of Fe^2+^ in large unidentified cell types (Figure 4B), suggesting that these Fe^2+^ intracellular patterns might be coupled with the animal’s bioenergetics and magnetoreception, potentially affecting mucus associated feeding behaviors which can be experimentally tested in future research. It might not be an overstatement that this research direction on placozoans could be relevant to better understanding systemic adaptations to microgravity and animal health under stress factors.

In sum, *Trichoplax*, in particular, and Placozoa, in general, can be viewed as prospective reference species in traditional evolutionary and system biology but have the yet unexplored potential for planetary ecology and space biomedicine (Romanova and Moroz, 2024). Additional long-term observations of population dynamics, and ideally at different geographic locations both in laboratory culture conditions and natural habitats are needed.

## Data Availability

The original contributions presented in the study are included in the article/Supplementary Material, further inquiries can be directed to the corresponding author.

## Supplementary material

SUPPLEMENTARY MATERIAL S1 Statistical analysis.

SUPPLEMENTARY TABLE S1 Original datasets for statistical analyses.

SUPPLEMENTARY TABLE S2 Mann-Whitney U-test by months for all investigated groups (CCL, ACL, DCL).

SUPPLEMENTARY FIGURE S1 Analysis of population growth data of *Trichoplax* with the ap index during 2024 (original file).

SUPPLEMENTARY FIGURE S2 Analysis of population growth data on placozoans.

SUPPLEMENTARY FIGURE S3 Comparisons of the present datasets with previously published data from (Maruyama, 2004).

SUPPLEMENTARY FIGURE S4 Comparison of standard plots (left) and sliding windows for 5 observations (right). The upper plot includes all groups of animals, CCL, control; ACL, ampicillin-treated animals; DCL, doxycyclin-treated animals.
