# Supplementary figures and images for: Long-term dynamics of placozoan culture: emerging models for population and space biology
Source: Front Cell Dev Biol. 2025 Jan 8;12:1514553. doi: 10.3389/fcell.2024.1514553 (PMC11751234; doi:10.3389/fcell.2024.1514553)

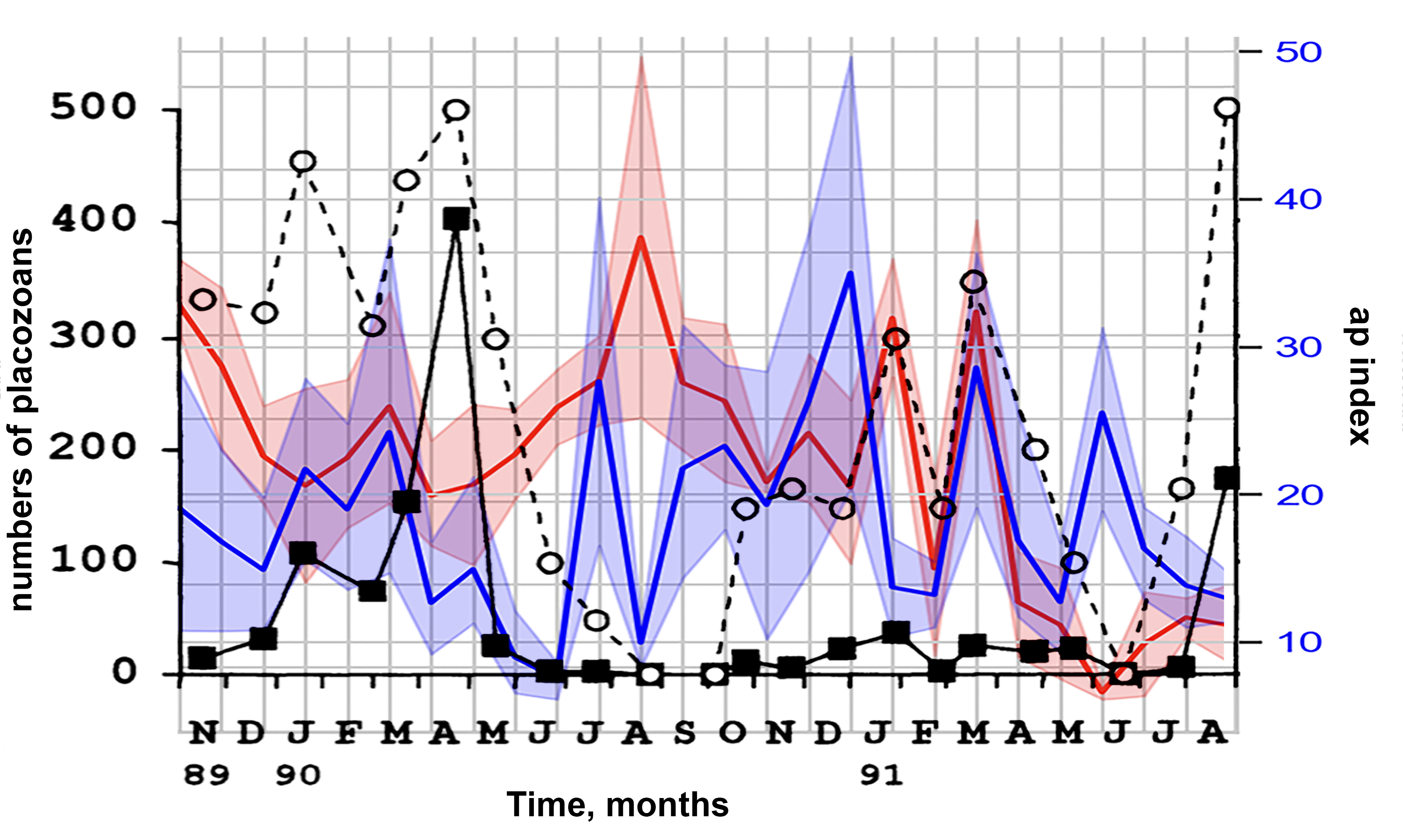

Supplement: Supplementary file 1 [file Image3.jpeg]

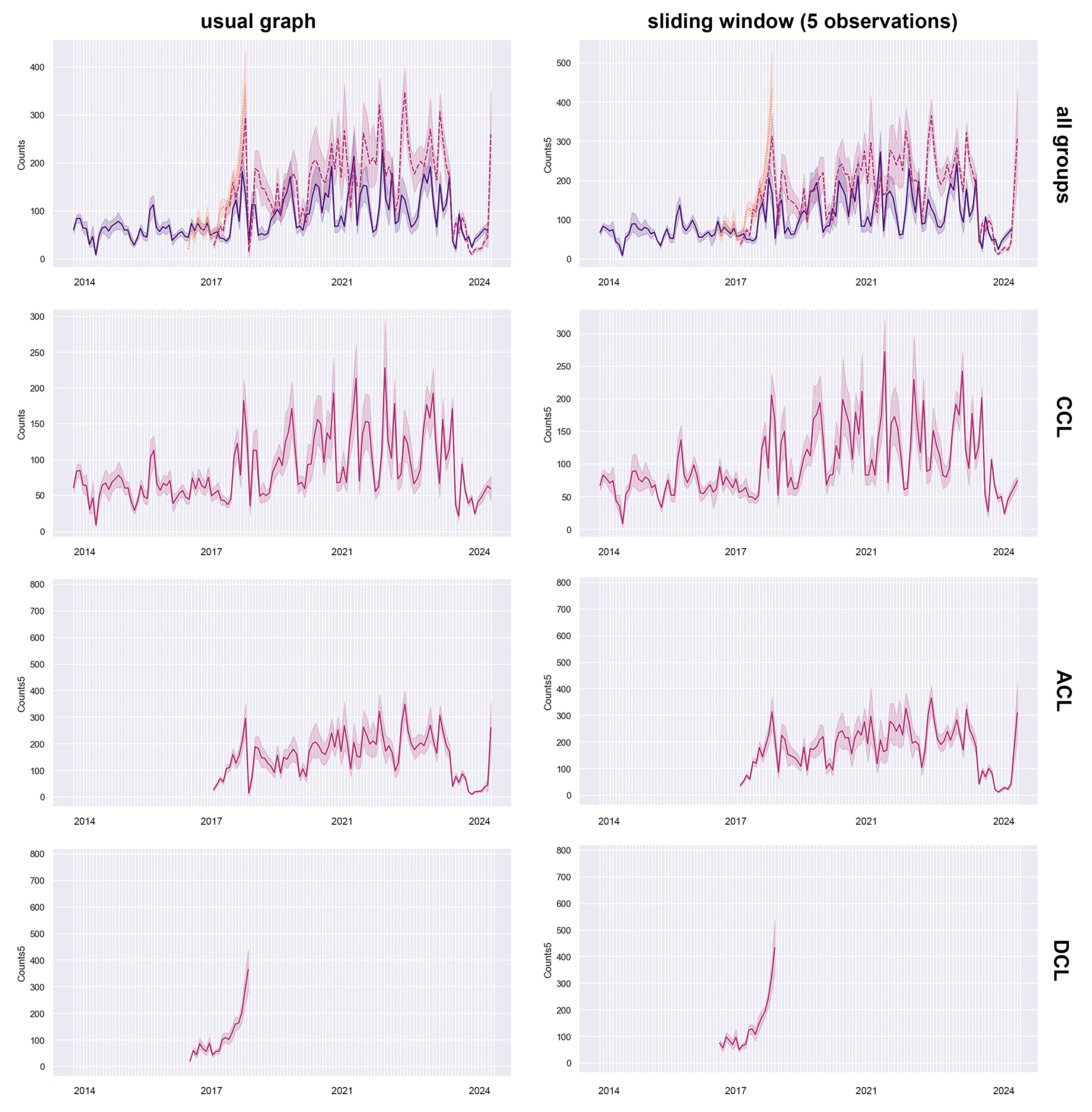

Supplement: Supplementary file 3 [file Image4.jpeg]

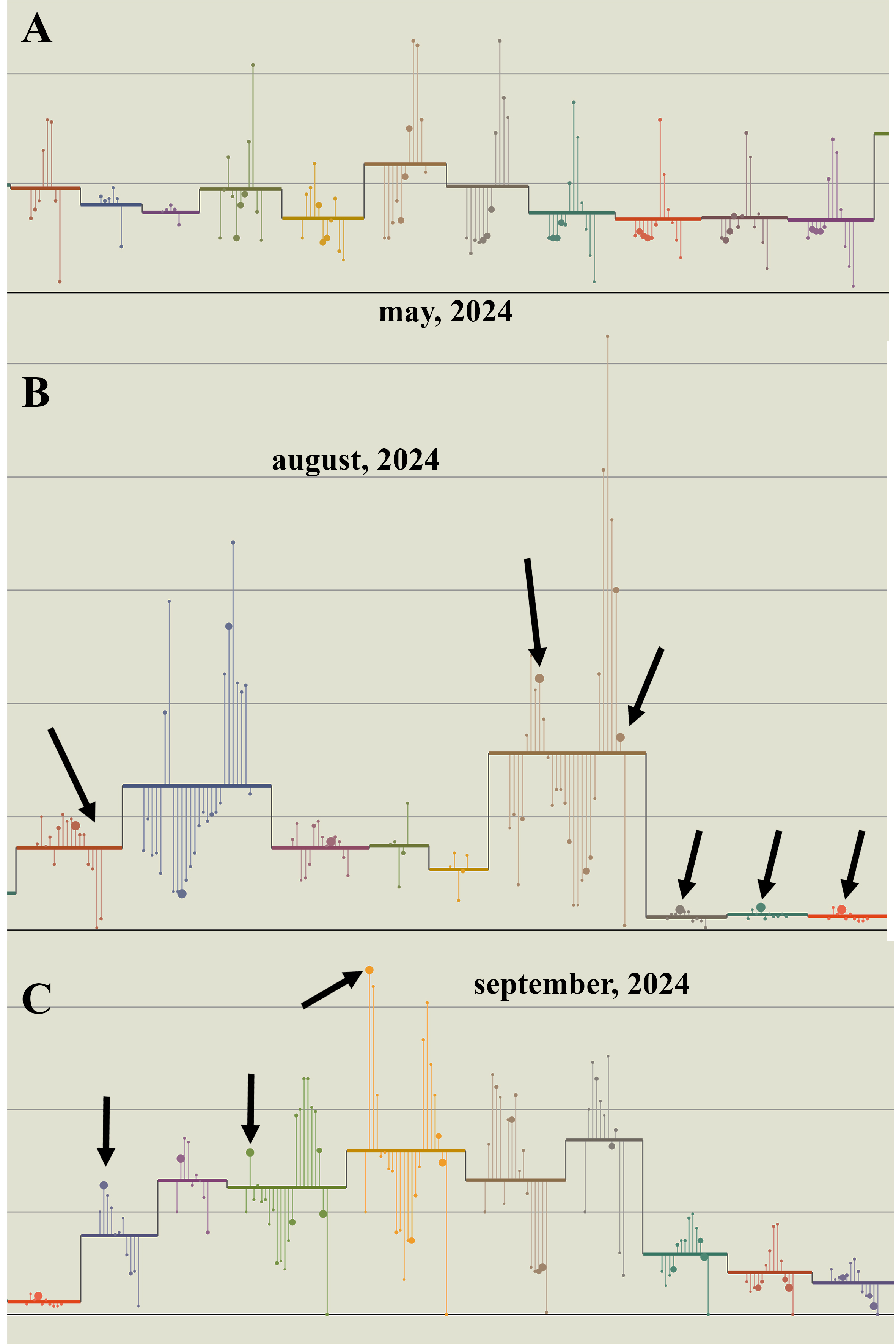

Supplement: Supplementary file 4 [file Image2.jpeg]
